# Effect of vaccination against COVID-19 spreading

**DOI:** 10.2183/pjab.97.027

**Published:** 2021-11-11

**Authors:** Hirotaka SUGAWARA

**Affiliations:** *1 KEK, Tsukuba, Ibaraki, Japan.

**Keywords:** SARS-CoV-2, COVID-19, vaccination, variant

## Abstract

We continue (Ref. 1: Proc. Jpn. Acad. Ser. B **97**, 22–49) to analyze the COVID-19 status. We concentrate on the following issues in this work:

1. Effect of vaccination against the spreading of SARS-CoV-2.

2. General landscape of the world situation concerning vaccinations.

3. Some aspects of the new variants of SARS-CoV-2.

Our findings include:

1. With vaccinations, it is fair to say that we have entered a new phase in the fight against the virus SARS-CoV-2. We have analyzed some preliminary data to find how vaccinations can be effective against COVID-19 spreading. This analysis is based on, and is a continuation of, our first paper quoted in Ref. 1.

2. If Tokyo (or Japan) continues to keep its vaccination schedule (starting in early April, 2021 and finishing it for elderly, 65 or older, in 4 months), it will see a sign of control of the virus in early June, 2021 although we see changes of this status due to new, more contagious variants.

3. The strength (parameter β) of a new contagious variant can be estimated based on the initial data on the variant (Section 5).

## 1 Introduction —Formalism to analyze possible vaccination effect—

The effect of vaccinations on COVID-19 spreading can be estimated by our formalism. We start from the standard SIR model,^2,3)^ and simplify it on one hand and generalize it on the other.^1)^

It goes as follows:

The SIR model is defined to be a set of differential equations for *S*, susceptible; *I*, infected and *R*, recovered:

dS/dt=−βIS/N, [1]

dI/dt=βIS/N−γI, [2]

dR/dt=γI. [3]

β in Eqs. [1] and [2] is the rate of decrease of the susceptible *S* and γ in Eqs. [2] and [3] is the recovery rate. We have *S* + *I* + *R* = *N*, where *N* is the total population.

We modify the model in the following way:

(1) Approximation.

At least in the case of COVID-19 we can safely assume that *S* ≫ *I*, *R*.

Therefore, we set

S/N≃1. [4]

The above equations then become linear:

dS/dt=−βI, [5]

dI/dt=βI−γI, [6]

dR/dt=γI. [7]

(2) Modification.

(2a) It is usually assumed that β and γ are independent of time. But this is not appropriate because β depends on social distancing; for example, and, therefore, it depends on time. The parameter γ can depend on time with increasing medical effort, although the natural recovery power is independent of time.

We define β as follows: it can be written as λ*f*^2^, where λ is the basic infection rate and *f*^2^ is the contact rate (*f*, itself, is the rate of the contacting population) of the population we wish to consider. Both parameters can depend on time. The time dependence of λ can come from a mixture of new variants, for example.

(2b) Another important input is the following: We divide *I* into two parts, with one being the infected population, but not so recognized (both symptomatic and asymptomatic infections are included). We write this as *I*_*u*_. The other component, *I*_*h*_, is the infected and segregated from uninfected population.

$$I=I_{u}+I_{h}.$$ [8]

*I*_*h*_ does not contribute to *I* in β*I* in Eqs. [1] and [2]. The equations become:

$$dS/dt=-\beta I_{u},$$ [9]

$$dI/dt=\beta I_{u}-\gamma I,$$ [10]

dR/dt=γI. [11]

The second of this set of equations (Eq. [10]) is important for any analysis. The first and the third equations are just the definitions of *S* and *R*.

*I*_*h*_ comes out of *I* by the PCR test, and assuming that the test ability can cover the entire population with a symptom, we have

$$dI_{h}/dt=\sigma I_{u}.$$ [12]

Equation [10] becomes,

$$\begin{array}{rl} & dI_{u}/dt+dI_{h}/dt\\ & \;=dI_{u}/dt+\sigma I_{u}=\beta I_{u}-\gamma (I_{u}+I_{h})\\ & \;=(\beta -\gamma )I_{u}-\gamma I_{h}. \end{array}$$ [13]

Substituting Eq. [12] to Eq. [13], we obtain,

$$\frac{dI_{u}}{dt}=(\beta -\sigma -\gamma )I_{u}-\gamma \int_{0}^{t}\sigma I_{u}dt.$$ [14]

This is an integro-differential equation for *I*_*u*_. When we can ignore the last term, we reach:

$$dI_{u}/dt=(\beta -\sigma -\gamma )I_{u}.$$ [15]

Equation [15] is what we use in our analysis. The last term in Eq. [14] turns out to be small because of the small value for γ, but should be considered eventually.

### Inclusion of effect of vaccination.

We can include the effect of (incomplete) artificial herd immunity (vaccinations, for example) by assuming *S* ≠ *N* to be as follows:

$$\frac{S}{N}=1-\frac{m_{i}}{N}\equiv 1-\phi ,$$ [16]

where *m*_*i*_ = number of those who acquired herd immunity or vaccination or some other cause to avoid infection. Then, the equation for *I*_*u*_ becomes

$$dI_{u}/dt=\beta I_{u}(1-\phi )-\gamma I_{u}.$$ [17]

Therefore, we must change β to

$$\beta^{\prime }=\beta (1-\phi ).$$ [18]

This leads to

$$\begin{array}{rl} & (\beta -\sigma -\gamma )\to (\beta^{\prime }-\sigma -\gamma )=(\beta (1-\phi )-\sigma -\gamma ),\\ & \;dI_{u}/dt=\kappa I_{u}=\{\beta (1-\phi )-\sigma -\gamma \}I_{u}. \end{array}$$ [19]

The daily infection number is given by

$$\frac{dI_{h}}{dt}=\frac{1}{\sigma }I_{u0}\exp\int_{0}^{t}\{\beta (1-\phi )-\sigma -\gamma \}dt.$$ [20]

Since the time dependence of ϕ is given artificially in the case of vaccinations, we must give up Eq. [1] or Eq. [5] in this case. In treating the natural herd immunity these equations can be used.

Here we assume that the natural herd immunity is small, and we can neglect its contribution.

## 2 Analysis of the vaccination effect when taking Israel as an example

Israel has been one of the fastest countries to start COVID-19 vaccinations. We now analyze the data of Israel regarding the vaccination.

The data (https://ourworldindata.org/vaccination-israel-impact) show, as of February 13, 2021:

Population rate of the first vaccination → 46%.

Population rate of the second vaccination → 25%.

According to U.S. FDA (https://www.fda.gov/media/144434/download), the effectiveness of each case is 80% and 95% for the Moderna vaccine. Precisely, the number is the prohibition rate of lightly symptomatic patients to develop severer symptoms, or the prohibition rate of asymptomatic infections to become symptomatic. It does not mean the rate of dis-infection. Nevertheless, we may interpret this as such to see if it agrees with the data.

This gives an estimation of ϕ, defined in Section 1, as

$$\begin{array}{rl} \phi & =0.25\times 0.95+0.21\times 0.80\\ & =0.401\text{ on February 13, 2021}. \end{array}$$

The vaccinations in Israel were started on December 19, 2020, and the total number increased almost linearly. This shows that, where taking *t* = 0 for December 19, 2020

$$\phi =\frac{0.401}{55}t=0.0073t.$$ [21]

The data of Israeli daily infections from January 15 to February 14, 2021 is given as (https://www.worldometers.info/coronavirus/country/israel/):

$$\begin{array}{rl} & (3235,8450,4833,9919,7380,10213,7027,6159,\\ & 4933,3442,8962,7213,8012,7305,5096,4798,\\ & 4646,8811,7732,8896,6744,5238,4727,3756,\\ & 7189,7191,6010,5083,3934,3100,2534). \end{array}$$ [22]

A least-squares fit to this histogram is given by

$$\exp[8.56112+0.0480911t-0.0019578t^{2}].$$ [23a]

**Figure 1.**
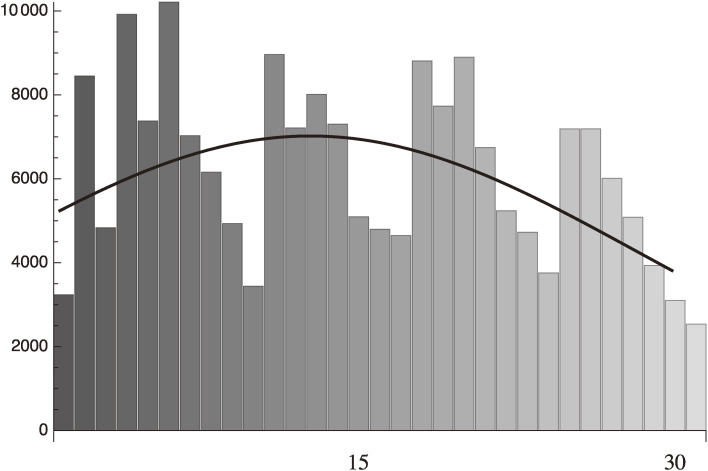
Fitting to the daily infection from January 15 to February 14, 2021 data of Israel with the standard deviation, 1864.87.

Equations [22] and [23a] are shown in Fig. 1 with the standard deviation being calculated as:

$$\text{sigma}=\sqrt{\{\frac{1}{31}\sum_{n=1}^{31}([22](n)-[23](n){)}^{2}}=1864.87,$$ [23b]

which gives,

sigma/average daily infection number=0.30 [23c]

The fairly large standard deviation given in Eq. [23b] is due to the artificial fluctuation of the data, which have a clear dependence on the day of the week when the data is taken. The fitting averages these artificial fluctuations.

The above fitting shows that

κ=β(1−ϕ)−σ−γ=0.0480911−0.0039t. [24]

Here, γ = 0.05, as is obtained from the data of the cruise ship Diamond Princess as shown in Ref. 1). β must be considered being independent of time because there was no substantial change in Israel of the social distancing policy during the above-mentioned period.

As for the value of σ, there are two options:

(1) We may take it to be vanishing; σ = 0, assuming the dominance of asymptomatic infection and there is no effort in Israel to search for asymptomatic infection by large-scale PCR testing.

(2) Leave the σ and calculate it from the above analysis. ϕ is defined as:

$$\phi =\frac{0.401}{55}(t+27)=0.0073(t+27).$$ [25]

This is when we just shift the origin from December 19, 2020 to January 15, 2021. We also have to consider that delay of vaccination becomes effective. Supposing that it is approximately one week, then we have

ϕ=0.0073(t+27−7)=0.0073(t+20). [26]

We discuss these two cases separately as follows:

(1) σ = 0 case.

$$\begin{array}{rl} \kappa & =\beta (1-0.0073(t+20))-0.05\\ & =0.0480911-0.0039t. \end{array}$$ [27]

We, therefore, get

(1−0.146)β−0.05=0.0480911, [28a]

0.0073β=0.0039. [28b]

From Eq. [28a] we get

β=0.115. [29a]

From Eq. [28b] we get

β=0.534. [29b]

These two numbers do not agree.

(2) σ ≠ 0 case.

(1−0.146)β−σ−0.05=0.0480911, [30a]

0.0073β=0.0039. [30b]

We then get

β=0.534, σ=0.36. [31]

This shows the importance of (A) a vaccination together with (B) the search and find method for decreasing the number of infections, at least from an analysis of data from Israel. In fact, the number of PCR tests in Israel is fairly large: (https://ourworldindata.org/coronavirus-testing). However we experienced some decrease during the period of the above analysis: 13/1,000 tests in early January and 8/1,000 on February 11, 2021.

We can conclude the following: If there were no vaccination, κ would become positive, and the daily infection must have been increasing during this period as follows:

$$\begin{array}{rl} \kappa (\phi =0) & =\beta -\sigma -\gamma =0.534-0.36-0.05\\ & =0.124. \end{array}$$ [32]

This clearly shows that the decrease was due to vaccinations.

The effective reproduction number, *R*_*t*_, is given by

$$\begin{array}{rl} R_{t} & =\frac{\beta (1-\phi )}{\sigma +\gamma }=\frac{0.534(1-0.0073(t+20))}{0.36+0.05}\\ & =1.112-0.009t. \end{array}$$ [33]

We have

$$R_{t}<1\;\text{for }t>12.4\text{ (January 29, 2021)}.$$ [34]

### Herd immunity.

In passing, we discuss possible herd immunity obtained by vaccinations.

We can define the quantitative index for herd immunity in the standard SIR model^2,3)^ as follows:

The herd immunity index is the percentage of a non-susceptible fraction of a given population which enables non-spreading of the disease without taking any other measures.

In other words, we can formulate it as follows:

$$R_{0}=\frac{\lambda_{0}(1-\phi )}{\gamma }<1,$$ [35a]

$$\phi >1-\frac{\gamma }{\lambda_{0}}=1-\frac{0.05}{\lambda_{0}}.$$ [35b]

We apply this formula to the known cases of λ_0_. In our previous paper^1)^ we calculated λ_0_ for Los Angeles, New York and Tokyo as follows:

$$\text{Los Angeles: }\lambda_{0}=0.726\to \phi >0.932,$$ [36a]

$$\text{New York: }\lambda_{0}=0.54\to \phi >0.908,$$ [36b]

$$\text{Tokyo: }\lambda_{0}=0.18\to \phi >0.72.$$ [36c]

This shows that the U.S. will not be safe until more than 90% of the population gets the vaccination. Japan may become safe if 72% of the population becomes vaccinated. The above values for λ_0_ do not include the effect of variants, which will make these values at least 30% larger, as will be shown later in this paper. The difference between the U.S. and Japan may be explained by the genetic difference and/or social behavior, but this is not our current concern. Until the necessary numbers are achieved we must combine the vaccination effort with either/both social distancing or/and broad PCR testing.

## 3 Application: What will happen to Tokyo after April, 2021? —Preliminary analysis—

In this section and the following sections, we use the symbol “*x*” rather than “*t*” to indicate the day variable when it is not confusing.

As an application of the above formalism concerning the vaccination effect, we examine the Tokyo case, which is of some global interest because Tokyo is trying to host the Olympic and Paralympic Games, starting in July, 2021. An assumption is made that the probability of preventing infections is about 90% or similar numbers, depending on the vaccines (https://www.fda.gov/media/144434/download). This assumption seems to work in the case of Israel, as is shown in the previous section. The study was based on the data that vaccinations are preventing the symptomatic patients from becoming severe symptomatic patients with a probability of 90%. Recent data also show that with the same percentage the vaccine is preventing asymptomatic patients from becoming symptomatic.

The latter case is somewhat interesting. Even if the vaccine cannot prevent the infection, 90% stay in the form of an asymptomatic status, which is nothing but the status of herd immunity or state of symbiosis with the virus.

This case is identical to the prevention of the infection, assuming that the announced daily infection numbers do not include asymptomatic infections, which seems to be the case in most countries.

Therefore, we can stick to the assumption verified in the Israel case that vaccination can prevent the infection, and it is universal in the case about which we are concerned: the case of COVID-19.

We first present a summary of an analysis of the Tokyo data:

(1) There were not so many changes in the social distancing situation since the time of phase 2 (which started towards the end of May, 2020) until early January, 2021, when the emergency was declared.

We could fit the data of the increasing period of phase 2^1)^ with

$$\beta =\lambda f^{2}=0.107,\;\gamma =0.05.$$ [37]

(2) In the decreasing period of phase 2 we needed to add

σ=α(1+0.035x). [38]

This comes from an increase of the PCR testing during the later period of phase 2 with α being a free parameter. The fitting of the data is given by

$$\frac{dI_{h}}{dt}=\exp\left(\int_{0}^{t}\kappa dx\right),\;\kappa =\beta -\sigma -\gamma$$ [39]

with above values for β, σ and γ. We take α to be 0.02 to fit the data of phase 2.

We can conclude that the decrease of the infection in the phase 2 was due to the increase of the PCR test numbers. There was no increase in social distancing during this period. This interpretation of a decrease due to the increase in the testing is probably unique to our analysis.

(3) Towards the end of phase 2 we observed a flattening of the PCR test numbers.^1)^

$$\begin{array}{rl} x_{s} & =\text{September 10, 2020}-\text{May 25, 2020}\\ & =108\text{ days}. \end{array}$$ [40]

The value of σ on this date is given by,

$$\sigma =\alpha (1+0.035x_{s})=3.78\alpha .$$ [41]

This shows that we can fit the later data (increasing period of phase 3 (October 20 to November 15, 2020)) by

$$\exp(A+0.0569139x-3.78\alpha^{\prime }x).$$ [42a]

Also, Eq. [42a] must be equal to the fitting curve given by

exp⁡(4.87812+0.0276343x). [42b]

We, therefore, obtain

$$\alpha^{\prime }=\frac{0.0569139-0.0276343}{3.78}=0.08.$$ [43]

By combining the above analysis of the (1) increasing period of phase 2, (2) the decreasing period of phase 2 and (3) the increasing period of phase 3, we obtain the following:

Phase 2. Increasing period:

exp⁡[2.08876+0.0569x], 0≤x≤63; [44]

Phase 2. Decreasing period:

$$\begin{array}{rl} & 8\exp[2.088+0.0569x-0.015(x+0.017x^{2}),\\ & \;64\leq x\leq 151; \end{array}$$ [45]

Phase 3. Increasing period:

$$\begin{array}{rl} & \exp[4.87812+0.0276343(x-152)],\\ & \;152\leq x\leq 284. \end{array}$$ [46]

**Figure 2.**
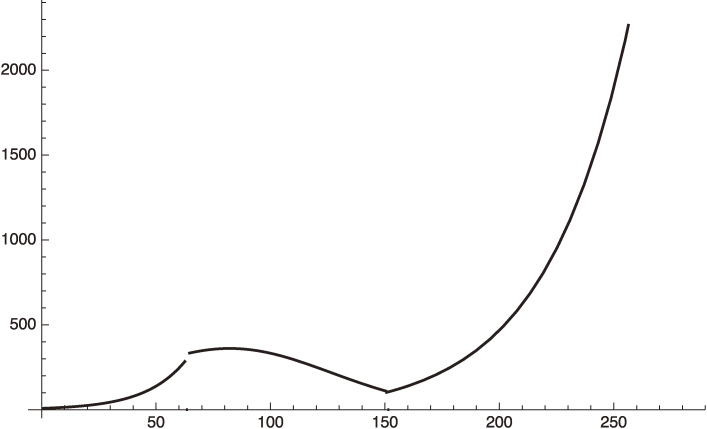
Phase 2 (increasing and decreasing period) and increasing period of phase 3. Here, *x* = 0 corresponds to May 10, 2020.

These curves are jointly shown in Fig. 2.

Next, an emergency was declared on January 8, 2021 and, as of February 18, 2021, it is still active. This means that *f*^2^ in β = λ*f*^2^ decreased during this period. The emergency(decreasing) period of phase 3 (ignoring the time dependence of *f*) can, therefore, be written as

$$\kappa =0.1069139f^{2}-0.05-0.008\times 3.78,$$ [47]

$$\begin{array}{rl} \frac{dI_{h}}{dx} & =\exp(A+(0.1069139f^{2}-0.05\\ & \;-0.008\times 3.78)(x-152)). \end{array}$$ [48]

**Figure 3.**
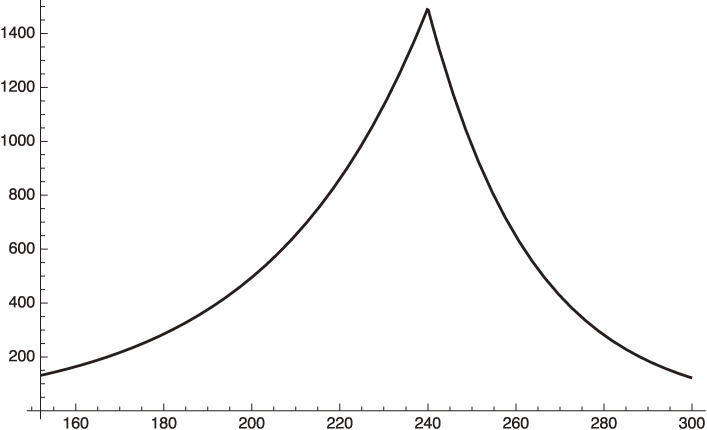
Phase-3 plot given by Eq. [48], where *f*^2^ = 1 or 0.36 for increasing or decreasing period respectively.

This equation should be used after January 8, 2021 (*x* = 240). We plot this in Fig. 3 for

$$f^{2}=0.36.$$ [49]

This value of *f*^2^ corresponds to assuming a reduction of the individual contact rate, *f*, by 40%.

Figure 3 shows that our estimate gives an excellent fit to the actual situation. After March 8, 2021 (*x* = 300), if the government relieves the emergency situation, we will be back to the early phase 3 parameters:

$$\begin{array}{rl} \kappa & =0.1069139f^{2}-0.05-0.008\times 3.78,\\ f^{2} & =1. \end{array}$$ [50]

**Figure 4.**
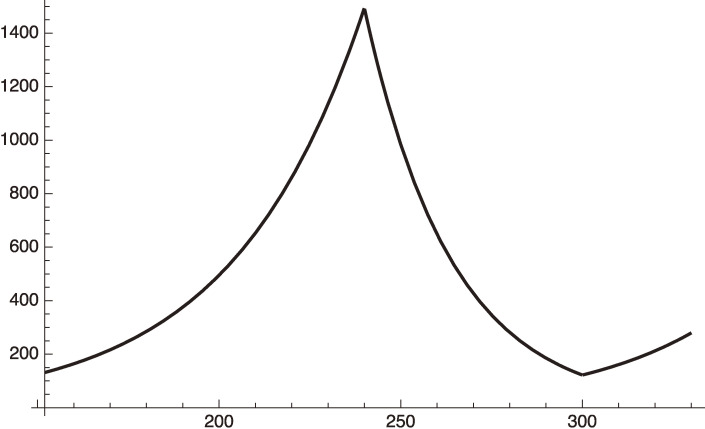
Increasing $\frac{dI_{h}}{dt}$ of Tokyo after March 8, 2021.

We plot this case in Fig. 4 by extending Fig. 3.

From the curve using Eq. [50] we get, for example,

$$\frac{dI_{h}}{dx}=121.965\;\text{for }x=300\text{ (March 8, 2021)},$$ [51]

and,

$$\frac{dI_{h}}{dx}=279.453\;\text{for }x=330\text{ (April 7, 2021)}.$$ [52]

### Effect of vaccination.

We now proceed to include the effect of vaccination which was supposed to start in early April, 2021 according to the current schedule. The current planning is that seniors over 65 years old will be vaccinated first, starting in early April, 2021, and, all of them will finish two vaccinations in 4 months. Then, the rest of the population will follow. Here, we assume a very optimistic number that the entire Tokyo population will be vaccinated within 100 days, starting in early April, 2021:

ϕ=0.01t. [53]

A more realistic number will be adopted later in Section 5 after taking the variant effect into account.

Including this contribution to Eq. [50], we obtain a formula for the daily infection number of Tokyo after April 8, 2021:

$$\begin{array}{rl} dI_{h}/dx & =2.042\exp[(0.0276343)(x-152)\\ & \;-0.1069139\times 0.005(x-330{)}^{2}], \end{array}$$ [54]

for the period of

330≤x≤400 (April 8 to June 7, 2021). [55]

**Figure 5.**
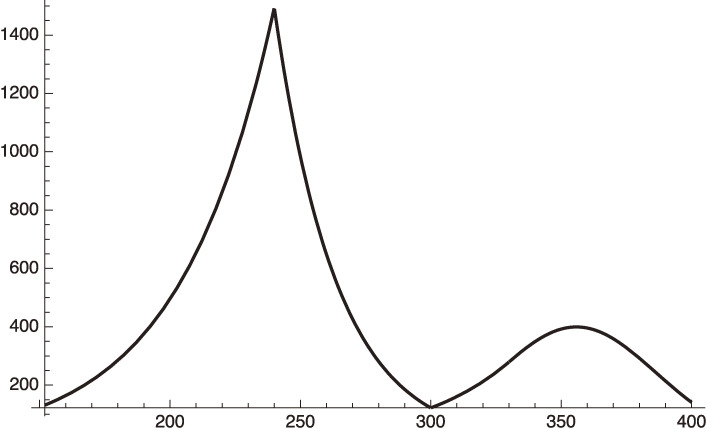
Behavior of daily infections of Tokyo, including the effect of vaccination after *x* = 330 (April 8, 2021).

We plot our predicted curve of phase 3 and phase 4 (after April 6, 2021) in Fig. 5.

Figure 5 shows that:

(1) After the end of the emergency period on March 7, 2021 (*x* = 300), the infection number will start to increase (122 on March 7 and 279 on April 8, 2021).

(2) After April 8, 2021 (*x* = 330) the effect of vaccination is included. The number will keep increasing until early May, 2021 (*x* ∼ 355), and will then start to decrease.

On June 7, 2021, the value of ϕ becomes,

ϕ=0.001t=0.6. [56a]

(1 − ϕ) is equivalent to *f*^2^ in the social distancing. This gives

$$f_{\text{equivalent}}∼0.63,$$ [56b]

corresponding to the 20% individual social distancing reduction since the time of phase 3 without the emergency declaration.

### Can Tokyo host the Olympic and Paralympic Games safely?

The above analysis shows that it is possible for Tokyo to host the Olympic and Paralympic Games as far as the Tokyo infection is concerned (with the optimistic assumption of perfect vaccination of the entire Tokyo population in 100 days after early April, 2021). One may still worry about a fourth peak which occurs around early May, 2021. To overcome this, we can suggest a few measures:

(1) Speeding up the vaccination.

If we multiply ϕ by a factor of 2, for example, we get the following curve:

$$\begin{array}{rl} \frac{dI_{h}}{dx} & =2.042\exp[(0.1069139-0.05-0.008\\ & \;\times 3.78)(x-152)-0.1069139\\ & \;\times 0.01(x-330{)}^{2}],\;\text{for }330\leq x\leq 400. \end{array}$$ [57]

**Figure 6.**
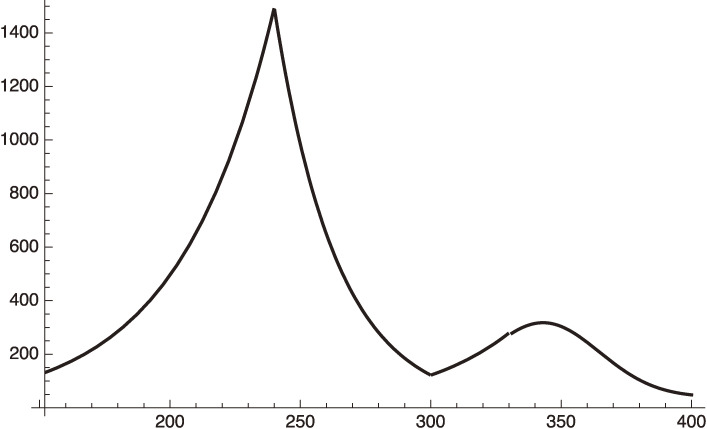
Tokyo daily infections when vaccination is done faster by a factor of 2 in Tokyo.

This is shown in Fig. 6.

Figure 6 shows that the peak position shifts from early May to late April, 2021, which is probably good to convince people that the pandemic is certainly under control, but maybe not enough to convince people that Games can be conducted.

(2) We may rather increase the number of PCR tests together with vaccinations. For example, multiplying a factor of 2 to α′ = 0.008 makes it 0.016. We thus have

$$\begin{array}{rl} \frac{dI_{h}}{dx} & =527.26\exp[(0.1069139-0.05-0.016\\ & \;\times 3.78)(x-152)-0.1069139\\ & \;\times 0.005(x-330{)}^{2}],\;\text{for }330\leq x\leq 400. \end{array}$$ [58]

**Figure 7.**
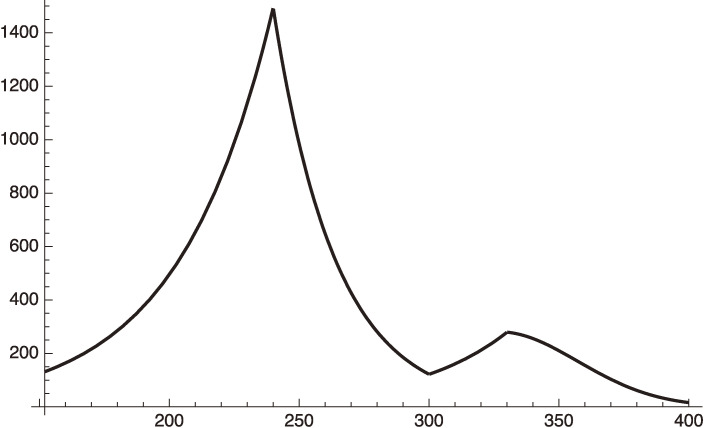
Tokyo daily infections with vaccination and PCR testing intensified by factor of 2.

This curve is shown in Fig. 7.

Figure 7 shows that the peak of the daily infection is shifted to early April, 2021 with a peak value of about 300/day just at the time of beginning vaccinations.

(3) One might think of extending the state of emergency until the vaccination begins. And then we follow the vaccination schedule.

In this case we get

$$\frac{dI_{h}}{dt}=34.85\;\text{for }x=330\text{ (April 7, 2021)}.$$ [59]

**Figure 8.**
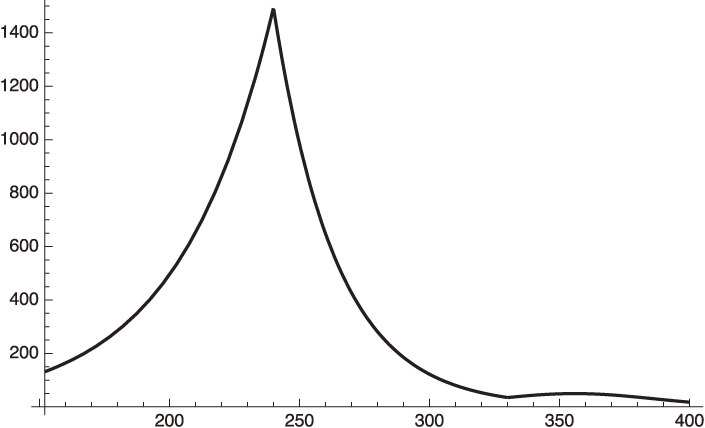
Daily infection number when the emergency is continued to April 8, 2021 (*x* = 330).

This is substantially smaller than 279.453 (Eq. [52]) for the case when the emergency declaration is lifted on March 7, 2021. We plot the curves for this case in Fig. 8.

Figure 8 shows that the fourth peak at around early May, 2021 is substantially suppressed.

### Tentative conclusion of Section 3.

As we have shown in the above analysis, vaccinations can make a difference in suppressing the spreading of SARS-CoV-2, including the possibility of symbiosis or herd immunity. If Tokyo starts the vaccination in early April, 2021, the pandemic will be under control by the end of June, and Tokyo can host the Olympic and Paralympic Games, although there will be a fourth wave of infections with its peak at around early May, 2021. The peak value of the daily infection is around 400.

If one wants to suppress this peak or wants to shift it to an earlier time, one can take several measures: (1) Faster vaccinations, (2) Enlarged PCR testing, (3) Emergency status (enforced social distancing) until vaccinations start.

*Among these measures*, (*1*) *seems improbable due to the fixed governmental schedule. Measure* (*2*) *seems to be rather easy: What we are proposing here in* (*2*) *is not large-scale unconditional PCR testing*, *but just the strengthening of the current effort to search for the origin of infections and*, *on the way*, *to perform PCR testing more widely. The proposal is to extend*, *by a factor of* *2*, *the testing to those without any symptoms*, *but have had contact with an infected patient.* (*3*) *results in many miseries among the least privileged people.*

If one wants to hold the Olympic and Paralympic Games safely as a symbolic event to show mankind’s victory over the SARS-CoV-2 virus, knowledge of the Tokyo status is certainly not sufficient. One must know the situation of all participating countries, which brings us to the next section.

## 4 Global status of vaccination against COVID-19

We start from our expression of the effective reproduction number, *R*_*t*_:

$$R_{t}=\frac{\lambda f^{2}(1-\phi (t))}{\sigma +\gamma }.$$ [60]

Here, λ stands for the basic infection rate, *f*^2^ is the herd social distancing rate, σ signifies the search-and-find method and γ is for the recovery rate.

If we have

$$R_{t}<1\;\text{with }f^{2}=1,$$ [61]

we can say that the virus is under control. In other words, if the infection number is decreasing without any enforcement on human activities, the disease will go away (or reach the status of symbiosis) sooner or later by keeping these parameters. A simplified version of Eq. [61] is

$$R_{t}=R_{00}(1-\phi (t)),$$ [62]

where *R*_00_ is the value of *R*_*t*_ at the time of the beginning of vaccinations.

Leaving the detailed description of the world-wide vaccination situation to the Appendix section, we now analyze the status of a few countries. For this purpose, we need to know parameters that appear in Eq. [60] or the parameters in the simplified Eq. [61]. We choose some highly populated countries from: (1) Africa, (2) North and South America, (3) Asia and (4) Europe. They are Nigeria and Ethiopia from Africa, the U.S. and Brazil from North and South America, China, India from Asia and Germany and France from Europe. Among them the analysis of the status of Nigeria, Ethiopia, Brazil and China is left to Appendix. Here, we analyze the situation of the U.S., India and France in some detail.

### U.S. status.

-Population: 328 m.

-National budget: U.S. $9.8 tn (2018).

-Current (as of February 22, 2021) effective reproduction number: *R*_0_ < 1.

-Vaccination status: Starting date depends on each state and we are not very clear about it. Tentatively we assume it is January 1, 2021. Then we have:

$$\phi =\frac{18.8}{100\times 52}t=0.0036t,$$ [63]

$$R_{t}=R_{00}(1-0.0036t).$$ [64]

This is to be identified with

$$R_{t}=\frac{\lambda f^{2}(1-\phi (t))}{\sigma +\gamma }.$$ [65]

Suppose β = λ*f*^2^ = time-independent and σ = 0 (corresponding to the case of dominant asymptomatic infection), we obtain

κ=β(1−0.0036t)−0.05. [66]

It seems that it is possible for the U.S. to see a substantial decrease in infections within one year. Nevertheless, herd immunity seems to be hard to achieve, as shown in Section 2 of the present work. It is possible that we may encounter a resurgence of COVID-19 in the U.S. sometime later in 2021.

We have analyzed this situation in more detail.

Data (https://ourworldindata.org/covid-vaccinations?country=USA) of the vaccination rate from January 2 to May 12, 2021 (every 10 days) is given as (number is the percentage of vaccinated (at least once) population):

$$\begin{array}{rl} \text{totally} & =\{1.3,2.8,4.9,7.9,10.6,13.3,16.1,\\ & \;21.0,25.6,31.0,36.8,41.4,44.8,41.9\}. \end{array}$$ [67]

This is least-squares fitted by a polynomial.

We find a good fit by going to at least a third-order polynomial:

$$3.75-2.0246x+0.797x^{2}-0.0317x^{3}.$$ [68]

**Figure 9.**
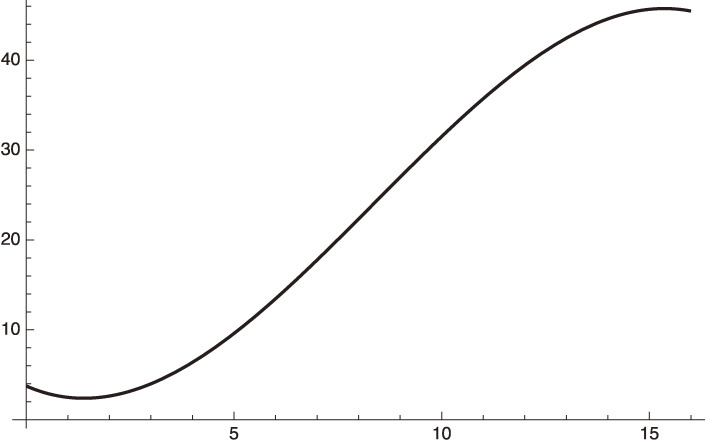
The U.S. total vaccination in population percentage from January 2 to May 12, 2021.

This is plotted in Fig. 9.

Figure 9 shows that the U.S. vaccinations will become saturated at around 50% of the total population no later than sometime in June, 2021.

At this specific time, we get

$$R_{0}=\frac{\lambda f^{2}(1-\phi )}{\sigma +\gamma }∼\frac{0.25f^{2}}{\sigma +0.05}.$$ [69]

Here, we used the value of λ ∼ 0.5, which we obtained for New York in our previous work.^1)^ This shows that we need at least *f* ∼ 0.5 or σ ∼ 0.2 to obtain an *R*_0_ of less than 1. Otherwise, there will be a resurgence of the infection number.

### Indian status.

-Population: 1.35 bn.

-National budget: U.S. $807 bn (2020).

-Current (as of February 22, 2021) effective reproduction number: *R* ∼ 1.

-Vaccination status: Covaxin, Covishield.

After early March, 2021, India is experiencing a huge explosion of COVID-19 spreading, probably due to new variants. Here, we analyze the situation in more detail. The data of the number of daily infections is given in https://www.worldometers.info/coronavirus/country/india/.

We use the data from April 1 to May 13, 2021:

$$\begin{array}{rl} & (81441,89019,92998,103793,96557,115269,126315,\\ & 131893,144829,152682,169914,160694,185248,\\ & 199569,216850,233943,260778,275306,256947,\\ & 294290,315802,332503,345147,349313,345531,\\ & 319435,362902,379459,386888,402110,392562,\\ & 370059,355328,382691,412618,414433,410326,\\ & 409300,366499,329517,348499,362406,348233) \end{array}$$ [70]

We fit the infection number in [70] by a least-squares method in the form of exp (polynomial). The result is:

$$dI_{h}/dx=\exp(11.111+0.1023x-0.0015x^{2}).$$ [71a]

$$\begin{array}{rl} & \text{Standard deviation/average infection number}\\ & \;=6.6\%. \end{array}$$ [71b]

**Figure 10.**
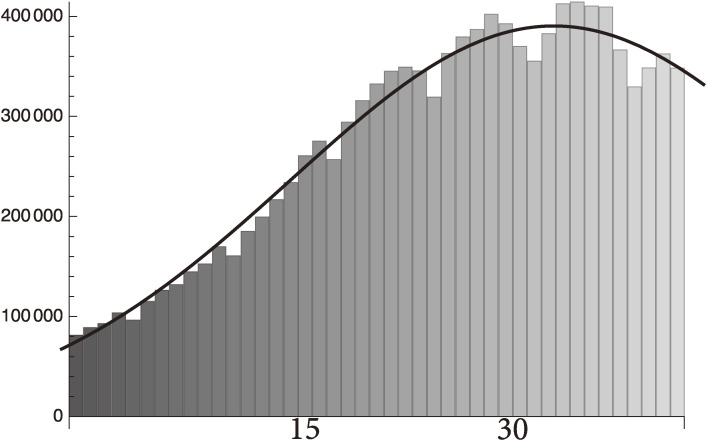
Fit to the infection number of India from April 1 to May 13, 2021 by the function in Eq. [71a].

This is far better than the case of Israel, showing that there are not many artificial weekly fluctuations in the case of India. The fit is shown in Fig. 10.

From Eq. [71a] we obtain

$$\kappa =\lambda (1-ax{)}^{2}-\rho (x)-\gamma =0.1023-0.0030x.$$ [72]

The only negative coefficient for the *x*^2^ term can come from ρ(*x*), meaning that the social distancing term, λ*f*^2^, cannot explain it. We put *a* = 0 for simplicity:

$$\begin{array}{rl} & a=0,\;\lambda -\rho (0)-\gamma =0.1023,\\ & \rho (x)=\rho (0)+0.003x. \end{array}$$ [73]

The meaning of the result is the following:

(1) The decline of daily infection after May 7, 2021 was due to the increase in test numbers. In fact, the test numbers in India are huge (https://coronaclusters.in/corona-testing-per-day-india): the total number up to May 13, 2021 was 311,324,100, and the single-day number was 1,875,515 on May 13, 2021. This is reflected in the above value of ρ(*x*).

(2) The contribution of social distancing together with vaccinations is revealed in making the value of λ*f*^2^ smaller:

λ=0.1523+ρ(0). [74]

The value of ρ(0) is to be calculated^1)^ using

$$\begin{array}{rl} \rho & =(\text{number of test cases per day}\\ & \;\times \text{positivity rate of the test})\\ & \;/(\text{number of infections}). \end{array}$$ [75]

Unfortunately, we are not aware of the positivity rate of the tests in India.

Vaccination in India reached almost 10% of the population as of May 13, 2021, which could contribute to making *a* in Eq. [72] smaller together with the social distancing, but it seems that they have not been significant up to now in reducing the daily infection number.

### French status.

-Population: 67 m.

-National budget: U.S. $1.6 tn (2020).

-Current (as of February 22, 2021) effective reproduction number: *R* ∼ 1.

-Vaccination status: The French case seems to be an easy one to work on since the *R* ∼ 1 period continued for some time, and we may assume that *R*_00_ ∼ *R* ∼ 1. Here, *R*_00_ is the effective reproduction number at the time of beginning vaccinations which we take to be January 1, 2021. From the value of vaccinations, 5.48/100 on February 21, 2021, we get,

$$\phi =\frac{5.48}{100\times 52}t.$$ [76]

Then, from Eq. [62], we get,

$$R_{t}=R(1-\phi )=1-0.001t.$$ [77]

It takes a few years to substantially reduce the infections or put it under the symbiotic situation without any substantial speeding-up of the vaccinations.

## 5 Some aspects of variants

### Preliminary considerations.

Several variants of SARS-CoV-2 have been reported so far from the U.K., South America, Brazil and India. We analyze some aspects of these variants in this section.

It is known^4)^ that the number of nucleotides of SARS-CoV-2 is approximately 30,000 and that the mutation rate is 23/year. Therefore, the probability of mutation for each nucleotide is 0.77 × 10^−13^ *N*_0_/day. Assume that one particular mutation leads to a new variant. The number of a particular variants created, denoted as *N*_*v*_, is at least in the initial stage given by

$$\begin{array}{rl} N_{\nu } & =\frac{N_{0}\times 0.77\times 10^{-3}}{30,000\times 360\text{ days}}\\ & =0.547\times 10^{-13}N_{0}/\text{day}, \end{array}$$ [78]

where *N*_0_ is the original (non-variant) virus number.

We obtain (assuming that the later stage increase of the variant is faster than the original one),

$$dN_{\nu }(t)/dt=0.547\times 10^{-13}A\exp(\beta_{\nu }t),$$ [79]

$$dN_{0}(t)/dt=A\exp(\beta_{0}t),$$ [80]

where β_ν_ or β_0_ is the effective infection rate for the original virus or the variant, respectively. We get

$$\begin{array}{rl} \frac{N_{\nu }365}{N_{0}365} & =\frac{\int_{0}^{365}\frac{dN_{\nu }(t)}{dt}dt}{\int_{0}^{365}\frac{dN_{0}(t)}{dt}dt}\\ & =0.547\times 10^{-13}\left(\frac{\beta_{0}}{\beta_{\nu }}\right)\exp(365(\beta_{\nu }-\beta_{0})). \end{array}$$ [81]

We calculated this result for a value of β_0_ = 0.107, taken from Eq. [37] for the Tokyo case.

We also assume that the new variant takes over the old one in year 50% to 90%: We obtain

$$\begin{array}{rl} 1\text{--}10 & =0.547\times 10^{-13}\left(\frac{\beta_{0}}{\beta_{\nu }}\right)\\ & \;\times \exp\left(365\times 0.107\left(\frac{\beta_{\nu }}{\beta_{0}}-1\right)\right). \end{array}$$ [82]

The solution is given by

$$\frac{\beta_{\nu }}{\beta_{0}}=1.79\text{--}1.86.$$

The fact is that the variants with a single mutation reported so far are comparable. (This is true for the variant 2021B.1.525 first found in the U.K. at around March, 2021 (https://www.cdc.gov/coronavirus/2019-ncov/cases-updates/variant-surveillance/variant-info.html).) The typical gene substitution of the spike protein is E484K. Our result shows that the value of β does not vary much whether the replacement percentage is 50% or 100%: β_*v*_ ∼ 1.8β_0_.

More precisely we have

$$\beta =\lambda f^{2}=\lambda (1-at{)}^{2}.$$

This shows that β is time-dependent. We must use λ rather than β:

$$\lambda_{\nu }=1.8\lambda_{0}.$$

This seems to show that the variants so far reported can increase the infection rate up to 80%, but not more. It is a different matter whether the vaccines so far invented work for the variants (https://www.statnews.com/2021/05/13/vaccines-work-variants-complicated/).

A variant originally found in India (delta type) has had two mutations, and was found to be more contagious.

In this case we have, instead of Eq. [82],

$$\begin{array}{rl} 1\text{--}10 & =\frac{(\frac{0.77\times 10^{-3}}{30,000}{)}^{2}}{360}\left(\frac{\beta_{0}}{\beta_{\nu }}\right)\\ & \;\times \exp\left(365\times 0.107\left(\frac{\beta_{\nu }}{\beta_{0}}-1\right)\right). \end{array}$$ [83a]

We obtain

$$\frac{\beta_{\nu }}{\beta_{0}}=2.26\text{--}2.32.$$ [83b]

We treat the delta variant case more phenomenologically later in this paper, while taking Tokyo case as an example.

### Application to Tokyo case.

We now apply the above result to the Tokyo case. This subsection treats only the variant studied in Section 3, which has a factor of 1.3-times larger infection rate compared to the original one. The case of the delta variant is treated in the next subsection.

The first variant in Tokyo was found early in January, 2021. Only after some periods we may see the effect of the new variant. We first consider when it will happen.

After January 8, 2021, we obtained a formula that provides a good fit to the data:

$$\kappa =0.1069139f^{2}-0.05-0.008\times 3.78,$$ [50]

together with

$$f^{2}=0.36.$$ [49]

This formula is plotted in Fig. 3.

We now add a contribution from the variant to this formula. The only difference is that the value of λ = 0.1069139 must be increased by around 30%, as is suggested by the above calculations, and its initiation is January 22, 2021.

(1) When λ is increased by 30%, we get

$$\begin{array}{rl} \frac{dI_{h}}{dx} & =\exp((0.13\times 0.36-0.05-0.008\\ & \;\times 3.78)(x-152))\\ & =\exp(-0.033(x-152)). \end{array}$$ [84]

This case is under control just as the original virus during this period.

(2) When λ is increased by 100%, we get

$$\begin{array}{rl} \frac{dI_{h}}{dx} & =\exp((0.22\times 0.36-0.05-0.008\\ & \;\times 3.78)(x-152))\\ & =\exp(-0.001(x-152)). \end{array}$$ [85]

Even this case is under control, meaning that daily infection number in Eq. [85] is still decreasing.

The above analysis shows that, although the mutation may occur and the ability of the virus to attach itself to ACE2 could be larger for the variants than the original virus, we can conclude that, at least as of March, 2021, the situation is under control due to the very effective social-distancing effect assumed in Eq. [49]. This is due to luck that new variants were found during the rather strict emergency situation.

To see what will happen under a different situation, we consider the case when *f*^2^ = 1 in Eq. [48], the total lifting of the emergency status.

Assuming that the variant has a 30% larger infection rate, we obtain

$$\begin{array}{rl} \frac{dI_{h}}{dx} & =K\exp((0.1069\times 1.3-0.05-0.008\\ & \;\times 3.78)(x-152)), \end{array}$$ [48]

where the normalization factor, *K*, must be determined, so that d*I*_*h*_/d*x* = 1 on March 21, 2021 (*x* = 314). We then get

$$\frac{dI_{h}}{dx}=0.0000706339\exp(0.059(x-152)).$$ [86]

The daily infection number is increasing, and we have

$$\frac{dI_{h}}{dx}=4,\text{ on April 7, 2021}.$$ [87a]

This is to be compared with

$$\frac{dI_{h}}{dx}=279,\text{ on April 7, 2021 for the original virus}.$$ [87b]

The value is not substantial, and the variant is far from dominating. If we have a variant with an infection rate factor of twice larger than the original one, we get

$$\frac{dI_{h}}{dx}=3.73539\times 10^{-10}\exp(0.134(x-152)).$$ [88]

Equation [88] implies that the infection rate factor of a twice larger does not lead to a catastrophic situation:

$$\frac{dI_{h}}{dx}\leq 10,\text{ on April 7, 2021}.$$ [87c]

As long as the vaccine works for the variant, we will be able to put the variant under control, even when the rate is a factor of twice larger than the original virus.

We have been treating the issue of variants in connection with the standard SIR model. We should be able to discuss this within the framework of the traditional population genetic model, which we will do in another paper to be published later, just to see the effect of any fluctuation, or that of neutral evolution^5)^ compared with the Darwinian evolution.

### Additional analysis of Tokyo situation.

The Section 3 and the above subsection have been devoted to the analyses of the Tokyo case while assuming that the vaccination will start in early April, 2021, which the Japanese government has claimed to do, and assuming that the entire Tokyo population will be vaccinated in 100 days, which is obviously too optimistic. The start of vaccinations in April, 2021 was also too optimistic. Moreover, none of the above three measures (1. speeding up the vaccinations, 2. intensifying the PCR test, 3. continuing the emergency status) have been taken seriously.

We can reanalyze the near-future situation of Tokyo by abandoning the assumption that it started vaccinations in early April, 2021, since we do not see any serious vaccination effort there. Rather, we assume that it started in early May, 2021. The vaccination rate is also changed to a realistic number, as will be shown below.

Another important factor is the new delta variant, which is much more contagious than the other SARS-CoV-2 virus found so far. Here, for simplicity, we treat the effect by a single parameter, $\eta =\frac{\beta_{\nu }}{\beta_{0}}\geq 1$ , while neglecting its time dependence after some time in June, 2021 where the 4th surge of daily infections had a minimum (specifically June 18, 2021). The actual variant ratio to the original one is, of course, time-dependent.

In our analysis, we had:

1. November 20, 2020 to January 10, 2021

$$\begin{array}{rl} & \exp[4.87812+0.0276343(x-152)],\\ & 152\leq x\leq 240. \end{array}$$ [89]

2. January 10 to March 8, 2021

$$\begin{array}{rl} & 58858\exp[(0.1069139\times 0.36-0.05-0.008\\ & \;\times 3.78)(x-152)],\;240\leq x\leq 300. \end{array}$$ [90]

3. March 8 to April 8, 2021

2.042exp⁡[0.0276343(x−152)], 300≤x≤330. [91]

4. April 8 to June 8, 2021

$$\begin{array}{rl} & 2.042\exp[(0.0276343)(x-152)-0.1069139\\ & \;\times 0.005(x-330{)}^{2}],\;330\leq x\leq 400. \end{array}$$ [92]

The last term in period 4 (0.1069139 × 0.005(*x* − 330)^2^) is the contribution of the vaccination, assuming that it was started on April 8, 2021 (*x* = 330).

If we change this to 0.1069139 × 0.005(*x* − 360)^2^, meaning that the vaccination was started in early May rather than April, 2021: The above 3 and 4 are changed to the following:

5. March 8 to May 8, 2021

2.042exp⁡[0.0276343(x−152)], 300≤x≤360. [93]

6. May 8 to July 8, 2021

$$\begin{array}{rl} & 2.042\exp[(0.0276343)(x-152)-0.1069139\\ & \;\times 0.005(x-360{)}^{2}],\;360\leq x\leq 420. \end{array}$$ [94]

**Figure 11.**
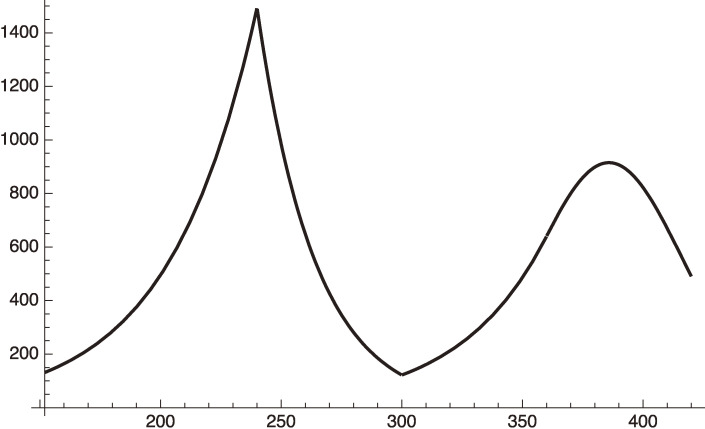
Daily infection number of Tokyo from March to July, 2021 assuming its vaccination is complete in 100 days starting in May, 2021.

These 4 formulae are plotted jointly in Fig. 11. But this figure is still optimistic, since we assumed that the entire Tokyo population would be vaccinated in 100 days:

ϕ=0.01t, t=x−360. [95]

A more realistic number comes from the fact that only 23.3% of the entire Tokyo population was vaccinated 4 months after early May, 2021. This gives

$$\phi =\frac{0.233\times 0.9}{120}=0.0017475.$$ [96]

In fact, the number of fully vaccinated population is about 18% and the one-time vaccinated is 13% on July 8, 2021. Assuming the linearity, we have

$$\phi =\frac{0.18\times 0.9+0.12\times 0.3}{60}t=0.0033(x-360).$$ [97]

Here, we assumed that one-time vaccination is 30% effective, whereas the two-time one is 90% effective. We adopt Eq. [97] in the following analysis. This is factor 3.0 smaller than Eq. [95].

From now on we concentrate on the daily infection number of Tokyo after May 8, 2021.

We must modify Eq. [94] with the new ϕ formula and this forces us to use non-unity *f*^2^, which may be logical, since the government declared the emergency status facing the 4th bump in the daily infection number.

We also take into account the contribution of the new delta variant after June 18, 2021, where the actual data starts to increase, most likely due to the delta variant. This is done by multiplying the parameter η > 1 by the original infection rate.

We replace Eq. [94] with

$$\begin{array}{rl} dI_{h}/dx & =\exp[A+(0.1069139f^{2}-0.05\\ & \;-0.0293)(x-152)-0.1069139f^{2}\\ & \;\times 0.0016(x-360{)}^{2}],\;360\leq x\leq 400. \end{array}$$ [98]

The formula after June 18, 2021 (*x* = 400) is modified to

$$\begin{array}{rl} dI_{h}/dx & =\exp[B+(0.1069\eta f^{2}-0.0793)(x-152)\\ & \;-0.1069\eta f^{2}\times 0.0016(x-360{)}^{2}],\\ & \;\;x\geq 400. \end{array}$$ [99]

We must use Eqs. [93], [98] and [99] to fit the data since March 8, 2021.

To obtain values for *A*, *f* and *B*, we need to fit the data between May 8 and June 18, 2021 and the data after June 18, 2021. We use a simpler method for choosing typical data samples to get these values rather than fitting the entire data (https://news.yahoo.co.jp/pages/article/covid19tokyo, in Japanese).

(a) For Eq. [98] (to determine *A* and *f*) we choose the following two data points to fix the parameters:

$$\text{for }x=360\text{ (May 8, 2021)},\;dI_{h}/dt\approx 1121,$$ [100]

$$\text{for }x=400\text{ (June 18, 2021)},\;dI_{h}/dt\approx 453.$$ [101]

We then obtain:

$$\begin{array}{rl} I_{h}/dx & =A+(0.1069139f^{2}-0.05-0.0293)(x-152)\\ & \;-0.1069139f^{2}\times 0.0016(x-360{)}^{2},\\ & \;\text{ for May 8 to June 18, 2021}, \end{array}$$ [102]

where we have

$$A=10.928,\;f^{2}=0.566075.$$ [103]

(b) For Eq. [99] (to determine *B* and η)

$$\text{for }x=400\text{ as above},\;dI_{h}/dt\approx 453,$$ [104a]

$$\text{for }x=440\text{ (July 28, 2021)},\;dI_{h}/dt\approx 3177.$$ [104b]

We obtain,

$$\begin{array}{rl} dI_{h}/dx & =B+(0.1069\eta f^{2}-0.0793)(x-152)\\ & \;-0.1069G\times 0.0016(x-360{)}^{2},\\ & \;\text{ for the period after June 18, 2021}, \end{array}$$ [105]

with

$$B=-13.0978,\;\eta f^{2}=1.48185.$$ [106]

We then get,

η=2.6177. [107]

**Figure 12.**
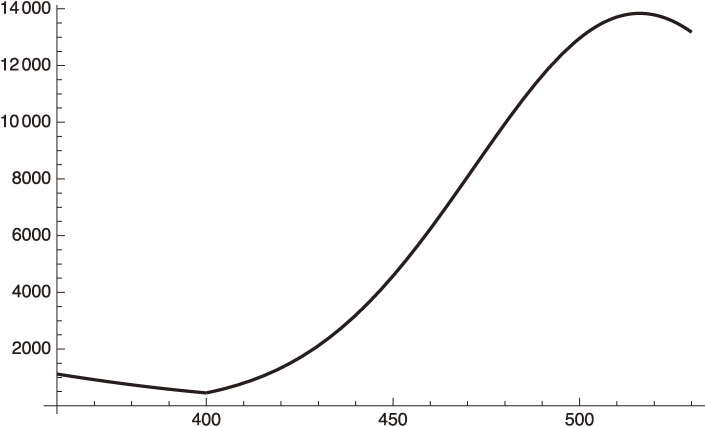
Daily infection number of Tokyo from May 8 (*x* = 360) to October 18, 2021 (*x* = 520).

Eqs. [102] and [105] are plotted in Fig. 12.

Figure 12 shows that the daily infection number will exceed 10,000 in early October, 2021. This is the case when the status of social distancing, the vaccination rate and the PCR test situation do not change. It starts to decrease early October, 2021 due to the vaccination increase if the current rate is maintained.

How can we avoid the above souring infection numbers in Tokyo? Since there is no way that Tokyo can substantially increase the testing numbers, if there is no substantial improvement of the social distancing in the city of Tokyo, there is only one way to avoid the above huge increase of the infection numbers until the end of September or early October, 2021 from exceeding 10,000 daily infections: increase the speed of vaccination.

Suppose that we increase the speed by a factor of 2 starting, for example, in late August (specifically August 28, 2021, *x* = 470), we have the formula for *dI*_*h*_/*dx* for *x* ≥ 470:

$$\begin{array}{rl} dn/dt & =\exp[-13.0978+(0.1069\times 1.482\\ & \;-0.0793)(x-152)-0.1069\times 1.482\\ & \;\times (0.0016(x-360{)}^{2}+0.0032(x-470{)}^{2}],\\ & \;\text{ after August 28, 2021}. \end{array}$$ [108]

**Figure 13.**
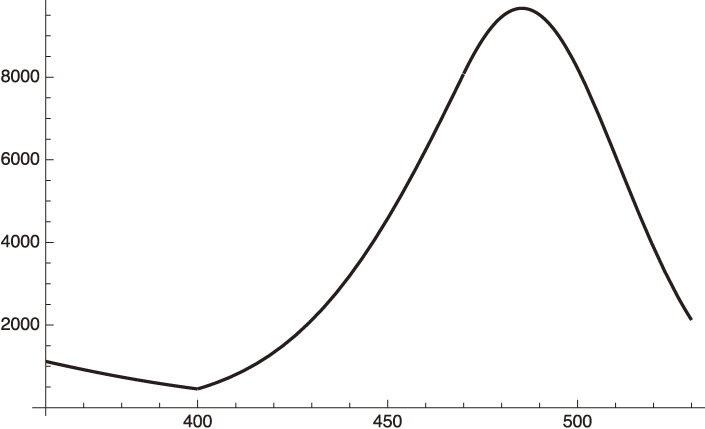
Daily infection number when the vaccination speed is twice faster after August 28, 2021 (*x* = 470).

Equation [108] is plotted in Fig. 13 together with the curves before August 28, 2021.

We see from Fig. 13 that the daily infection number is suppressed below 10,000, and starts to decrease at the end of September, 2021.

## 6 Conclusion

We investigated the COVID-19 status as a continuation of our previous work^1)^ within the framework of the standard epidemiology model: the SIR model. The model becomes linear with the assumption that the entire population is susceptible. A new parameter σ is introduced by the following equation:

$$dI_{h}/dt=\sigma I_{u}.$$ [12]

This means that the increase of the official daily infection number is proportional to the infected population at large, including the asymptomatic infections.

It is possible to formulate an evaluation of the effect of the vaccination rate within our modified SIR model, as is explained in Section 1.

We investigated the case of Israel, since it is one of countries that started vaccinations early. The model finds that vaccinations have a substantial effect in reducing the infections, as the data seem to show.

In order to completely eliminate the SARS-CoV-2 virus, we need to reach the level of herd immunity. We investigated this level by taking New York, Los Angeles and Tokyo as examples. It turned out that at least 90% of the population must be vaccinated in the former two cases and 70% in the example of Tokyo.

We then investigated (in Sections 3 and 5) the Tokyo situation in the near future based on past data analysis.^1)^ It depends crucially on when and how fast the vaccination process is performed and what sort of variant is infecting the population.

In Section 4 we investigated the world-wide status of the vaccination and, in particular, some of the most populated countries.

Among these countries, the U.S. vaccination, which started in early January, 2021, has already showed a sign of flattening the vaccination numbers way before it reaches herd immunity. It is quite possible that it will see some resurgence of COVID-19 unless some appropriate measures are taken.

India has been experiencing a sharp increase of the infection numbers starting sometime in March, 2021, perhaps due to a new powerful variant, but we see some indication of an end of the hardest time in May, 2021, mostly due to its large-scale testing activities.

In Section 5 we investigated how much stronger a given variant can become, by taking the case of B.1.525 as an example. B.1.525 was first found in the U.K. and the typical substitution of the spike protein is E484K. We found that the basic infection rate could be 1.3-times larger than the original one, but not more.

We then analyzed the Tokyo case with the possible delta variant taken into account. It seems that, as of July, 2021, vaccination rate of Tokyo cannot prevent the daily infection number to rise to over 10,000 sometime in late September, 2021 unless some measures are taken, although it will start decreasing sometime in October, 2021 owing to vaccinations. We found that it can be reduced to under 8,000 if the vaccination rate is doubled.

## Figures and Tables

**Table 1.** Status of various vaccines

Vaccine name*	Type**	Effectiveness	Storage
Pfizer-BioNTech	mRNA ($33)	95% protection from severity	−70 ℃
Moderna	mRNA ($20)	95%	−20 ℃
Oxford U. AstraZeneca	genetically modified virus ($4)	62–90%	regular fridge
Sputnik V	viral vector (traditional) ($7.5)	92%	regular fridge

CoronaVac (Chinese)	viral vector (traditional)	91% (in Turkey)	regular fridge
SinoVac (Chinese)	viral vector (traditional)	?	regular fridge
SinoPharm (Chinese)	viral vector (traditional)	80%	regular fridge
Covaxin (Indian)	viral vector (traditional)		
Covishield (Indian)	viral vector		

*The information of Indian vaccine is from https://www.bbc.com/news/world-asia-india-55748124.

**Price information is from https://www.bbc.com/news/world-asia-china-55212787.

## Appendix

### World-wide situation of the vaccination against COVID-19.

(1) Available vaccines.

Table 1 summarizes the situation.

(2) What is WHO doing on the vaccine issue? — COVAX program (https://www.who.int/initiatives/act-accelerator/covax) offers:

-Doses for at least 20% of countries’ populations.

-Diverse and actively managed portfolio of vaccines.

-Vaccines delivered as soon as they are available.

-Ending the acute phase of the pandemic.

-Rebuilding economies.

G7 decided to contribute $7.6 bn to help the COVAX program.

According to World Bank (https://blogs.worldbank.org/opendata/september-2020-global-poverty-update-world-bank-new-annual-poverty-estimates-using-revised), “43.6 percent (of world population) lived on less than $5.50 a day in 2017”.

They can hardly afford the mRNA vaccine by their 1-week income. So, it is appropriate to define poor countries with this definition.

We then get:

Poor population=0.436×7.8bn=3.4bn,
[A1]

20% of the above number is 0.68\,bn.
[A2]

If WHO wants to take care of this population,

(a) Pfizer vaccine costs

0.68bn×$33=$22.4bn;
[A3]

(b) Moderna vaccine costs

0.68bn×$20=$13.6bn.
[A4]

These numbers show that WHO cannot help poor countries with mRNA vaccines, which rich countries already have in use starting in January, 2021.

(3) Rich countries had already started or had a fixed schedule of the vaccination in early January, 2021, but how about poor countries, if they cannot afford mRNA vaccine?

(A) Countries signed up for Chinese vaccines: Singapore, Malaysia, Philippines, Indonesia, Turkey, Brazil, Chile, Arab Emirates, and Bahrain.

(B) Countries signed up for Indian vaccines: Bhutan, Maldives, Bangladesh, Nepal, Myanmar and Seychelles.

(4) We have the following statistics on global vaccination status (https://ourworldindata.org/covid-vaccinations).

Countries that have the vaccination (as of February 21, 2021, case number/100): Israel: 85, United Arab Emirates: 56, U.K.: 26.8, U.S.: 18.8, Bahrain: 16.16, Chile: 15.1, Serbia: 14.5, Turkey: 7.8, Poland: 7.17, Morocco: 6.67, Spain: 6.28, Germany: 6.05, Italy: 5.8, France: 5.48, Canada: 3.72, Brazil: 3.27, Bangladesh: 1.26, India: 0.8, Indonesia: 0.72.

### Status of most populated countries in four continents: Nigeria, Ethiopia, Brazil, China and Germany.

*Nigerian status*.

-Population: 177 m.

-National budget: U.S. $35 bn (2020).

-Current (as of February 22, 2021) effective reproduction number: *R*_0_ < 1.

-How costly is the vaccination?

For Pfizer vaccine, 177 m × $33 = $5.8 bn: more than 10% of national budget which makes it practically impossible for Nigeria to get Pfizer vaccine.

-Government is starting the talks with China on vaccine (https://www.aa.com.tr/en/africa/nigeria-in-talks-with-china-on-covid-19-vaccine/2099479).

*Ethiopian status*.

-Population: 109 m.

-National budget: U.S. $8.9 bn (2020).

-Current (as of February 22, 2021) effective reproduction number: *R*_*t*_ > 1.

In early April, 2021, daily infection number started to decrease meaning *R*_*t*_ < 1. The cause is unclear. Vaccination stays low (∼2 m).

-How costly is the vaccination?

For Pfizer vaccine, 109 m × $33 = $3.6 bn: more than 30% of national budget which makes it impossible for Ethiopia to get Pfizer vaccine. COVAX offered 2.2 m AstraZeneca vaccine.

-China is offering vaccine to Ethiopia (https://www.aa.com.tr/en/africa/china-offers-covid-19-vaccines-to-ethiopia-algeria/2141626).

*Brazilian status*.

-Population: 210 m.

-National budget: U.S. $611 bn (2020).

-Current (as of February 22, 2021) effective reproduction number: *R*_*t*_ ∼ 1.

-Vaccination status. Brazil approved AstraZeneca and SinoVac vaccines (https://www.bbc.com/news/world-latin-america-55699535).

*Chinese status*.

-Population: 1.39 bn.

-National budget: U.S. $5.4 tn (2020).

-Current (as of February 22, 2021) effective reproduction number: no data.

-Vaccination status: CoronaVac, SinoVac, SinoPharm.

*German status*.

-Population: 83 m.

-National budget: U.S. $2.04 tn (2020).

-Current (as of February 22, 2021) effective reproduction number: *R*_*t*_ < 1.

-Vaccination status: It is not proceeding as planned.
